# A Unified Microstructure-Based Constitutive Model for a Ni-Based Superalloy and Its Application in the Forging Processes of Disk

**DOI:** 10.3390/ma18112526

**Published:** 2025-05-27

**Authors:** Ning-Fu Zeng, Yong-Cheng Lin, Shu-Xin Li, Yun-Han Ling, Jin Yang, Ming-Song Chen, Hong-Wei Cai, Zi-Jian Chen, Gui-Cheng Wu

**Affiliations:** 1 School of Mechanical and Electrical Engineering, Central South University, Changsha 410083, China; 2 State Key Laboratory of Precision Manufacturing for Extreme Service Performance, Changsha 410083, China; 3 Rongcheng Huadong Metal-Forming Machinery Co., Ltd., Rongcheng 264300, China; 4 School of Mechanical Engineering and Mechanics, Ningbo University, Ningbo 315211, China; 5 China Academy of Machinery Beijing Research Institute of Mechanical & Electrical Technology Co., Ltd., Beijing 100083, China; 6 Guizhou Anda Aviation Forging Co., Ltd., Anshun 561005, China

**Keywords:** constitutive model, Ni-based superalloy, integrated computational framework, forging process

## Abstract

This study proposes a novel unified constitutive model that systematically integrates the microstructure evolution and macroscopic stress–strain response during the hot deformation of a Ni-based superalloy. The proposed model incorporates a suite of microstructural variables, including damage fraction, recrystallization fraction, δ phase content, average grain size, and dislocation density. Furthermore, the model explicitly considers critical macroscopic stress state parameters, specifically the magnitude and orientation of maximum principal stress, hydrostatic stress component, and Mises equivalent stress. A comparative analysis of rheological curves derived from uniaxial tension and compression experiments reveals that the prediction errors of the proposed model are less than 3%. The model is subsequently implemented to investigate the evolution characteristics of the damage accumulation fraction and δ phase content under varying stress directions and initial δ phase contents. An advanced computational framework integrating the finite element method with the proposed constitutive model is established through customized subroutines. The framework exhibits exceptional predictive accuracy across critical regions of disk forging, as evidenced by a close agreement between computational and experimental results. Specifically, the relative errors for predicting recrystallization fraction and average grain size remain consistently below 8% under varying stress–strain conditions. Testing results from four representative regions demonstrate a good alignment of high-temperature tensile properties with the macroscopic stress–strain distributions and microstructure characteristics, thereby confirming the model’s reliability in simulating and optimizing the forging process.

## 1. Introduction

Nickel-based superalloy disk forgings are widely employed in advanced engines due to their exceptional mechanical properties [1,2]. However, the processing of superalloys is inherently challenging, primarily due to their high recrystallization temperature [3,4], slow recrystallization rate [5,6], complex multiphase microstructure evolution [7,8,9], and significant deformation resistance [10,11,12]. These factors collectively impose significant constraints on the forging process. Constitutive models have emerged as indispensable tools for predicting material flow behavior and elucidating microstructure evolution in nickel-based superalloys during hot deformation [13]. Such models offer critical theoretical insights for optimizing processing parameters in industrial applications [14]. Therefore, the development of a unified constitutive model that integrates the macroscopic stress states and microstructure evolution during the hot forming of nickel-based superalloys is imperative. This advancement would significantly enhance the understanding and control of the forging processes for high-performance nickel-based superalloy disks.

The prevalent methodologies for constructing material constitutive models can be broadly classified into three main categories [15,16]: (1) fitting traditional phenomenological constitutive equations [17,18,19]; (2) developing models based on the intrinsic mechanisms governing macro-micro responses of materials, such as physical-based models [20,21,22]; (3) leveraging machine learning techniques that directly utilize measured flow stress data [23,24,25]. Widely used phenomenological models, including the Johnson–Cook model, Zerilli–Armstrong model, and Arrhenius models, are advantageous due to their simplicity, fewer parameters, and ease of implementation [26,27,28,29,30]. However, these models often suffer from limited predictive accuracy. On the other hand, machine learning-based models, such as those employing artificial neural networks [31,32] or statistical learning methods [33,34], exhibit superior accuracy in predicting material behavior. Nevertheless, their application is often restricted to macroscopic deformation parameters due to challenges in interpretability and the requirement for extensive datasets [35]. Conversely, the physical mechanism-based models are capable of effectively characterizing microstructure evolution in material [36]. As a result, they offer enhanced capabilities for controlling and optimizing forging performance, particularly in demanding industrial applications.

Scholars have developed several constitutive models considering intrinsic physical mechanisms of nickel-based superalloys. Li et al. [37] created a yield strength prediction model considering precipitates, grain boundaries, and solid solutions. Liu et al. [38] modeled tensile rheological behavior based on dislocation density and grain size, while He et al. [39] focused on μ phase evolution for compression rheological behavior. These models primarily focus on the rheological behavior of superalloys under uniaxial stress. Yan et al. [40] studied the dynamic recrystallization (DRX) behavior of a GH4720Li superalloy microalloyed with 0.3Y during hot compression and formulated the constitutive models to simulate the flow features and DRX behavior.

**Figure 1:**
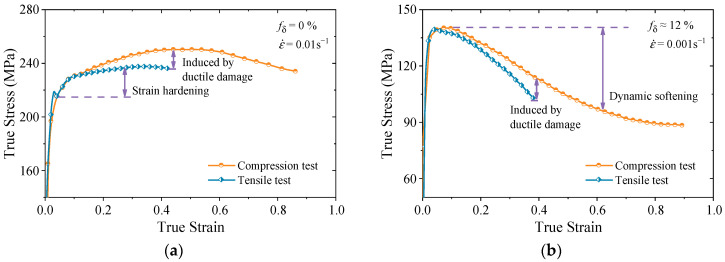
Typical rheological curves in uniaxial tension and compression tests [42,43]: (**a**) *T* = 950 °C; (**b**) *T* = 980 °C.

However, as illustrated in Figure 1, the rheological behavior of materials under tensile stress exhibits significant differences compared with that under pure compressive stress states. This divergence arises primarily due to the presence of plastic damage, which distinctly influences both the hardening and softening stages of material deformation. Consequently, it can be inferred that their microstructural evolution also varies. In actual deformation processing, the stress state of the material is inherently complex and variable [41], highlighting the necessity of developing a unified constitutive model that incorporates the influence of stress states.

The application of established physical constitutive models demonstrates their practical values better. Zhang et al. [44] integrated finite element (FE) methods to verify the physical mechanism model in extrusion processes and further optimized the process. Li et al. [31] predicted the yield strength of turbine disc forgings by employing a neural network model in conjunction with an analysis of physical mechanisms. However, there are limited reports on the application of models for macro-micro evolutions that occur during the forging process of entire Ni-based superalloy forgings.

To address the limitations identified in the existing research, a novel microstructure-based unified constitutive model, considering the effects of macroscopic stress–strain states, is developed in Section 2. The accuracy and reliability of this model are rigorously validated in Section 3. In Section 4, an integrated computational framework is proposed, which combines the developed constitutive model with the FE method. The feasibility of this framework is demonstrated through a practical case involving disk forging. Finally, in Section 5, the coupled relationships between the stress state and microstructure evolution at critical positions of disk forging are systematically analyzed based on the computational results. The mechanical properties of different regions are further evaluated through tensile tests, underscoring the practical significance and applicability of the proposed model in industrial settings.

## 2. Development of a Constitutive Model

### 2.1. Constitutive Law

By incorporating material damage theory [45] and rheological principles [46], the true stress *σ* can be expressed as follows:

(1) $$\sigma =\left(1-D\right)\left(\sigma_{t}+\sigma_{i}+\sigma_{g}+\sigma_{\delta }\right)$$

where *D* is the damage volume fraction, and $\sigma_{*}$ are stress contributions that influence the rheological behavior.

$\sigma_{t}$ is the thermal stress primarily arising from the thermal activation energy required for dislocations to move through the lattice or overcome short-range obstacles [47]. This stress component is generally expressed as a function of temperature (*T*) and plastic strain rate (${\dot{\varepsilon }}_{p}$), i.e.,

(2) $$\left\{\begin{array}{l} \sigma_{t}=\sigma_{0}\exp\left(\alpha_{t}f_{\delta }\right){\dot{\varepsilon }}^{n}\\ \alpha_{t}=\alpha_{t0}\exp\left(\frac{Q_{t}}{RT}\right),n=n_{0}\exp\left(-\frac{Q_{n}}{RT}\right) \end{array}\right.$$

where $\sigma_{0}(\mathrm{MPa}),\alpha_{t0}(s),n_{0},Q_{t}(\mathrm{kJ}/\mathrm{mol}),Q_{n}(\mathrm{kJ}/\mathrm{mol})$ are material constants. R is the universal gas constant (8.314 J·mol^−1^·K^−1^).

$\sigma_{i}$ is the stress resulting from the interaction of dislocations during plastic deformation [48]

(3) $$\sigma_{i}=M\alpha_{i}\mu b\sqrt{\rho }$$

where M (value = 3.06) is the Taylor coefficient in Face-Centered Cubic (FCC) metals, ρ means the average dislocation density with the initial value of 10^12^ m^−2^, $\alpha_{i}$ (value = 0.3) is a dislocation interaction constant [43], *b* (value = 2.54 × 10^−10^ m) represents the Burger’s vector, and *μ* (GPa) is the shear modulus that varies with temperature, which can be expressed as follows [49]:

(4) $$\mu =82.38-0.015T-5.5\times 10^{-6}T^{2}$$

In accordance with the Hall–Petch equation [50], micrometer-scale grains possess a strengthening effect, which can be expressed as follows:

(5) $$\sigma_{g}=\alpha_{g}\mu \frac{\sqrt{b}}{\sqrt{d}}$$

where $\alpha_{g}$ (value = 120.9) is a material constant [51], *d* means the average grain diameter.

The precipitation strengthening effect, specifically the δ phase bypass shear mechanism [43], refers to the additional stress required for dislocations to bypass δ phase precipitates distributed within the matrix. Consequently, the stress component $\sigma_{\delta }$ can be represented by the following:

(6) $$\left\{\begin{array}{l} \sigma_{\delta }=\alpha_{\delta }M\mu \sqrt{f_{\delta }}\frac{b}{r_{\delta }}\\ \alpha_{\delta }=\alpha_{\delta 0}\exp\left(\frac{Q_{\delta }}{RT}\right) \end{array}\right.$$

where $\alpha_{\delta 0}$ and $Q_{\delta }(\mathrm{kJ}/\mathrm{mol})$ are material constants, $f_{\delta }$ and $r_{\delta }$ represents the average volume fraction and equivalent grain size of δ phase, respectively.

The incremental relationship between the stress and strain rate follows the Hooke’s law [44]:

(7) $$\left\{\begin{array}{l} \dot{\sigma }=E\left({\dot{\varepsilon }}_{t}-{\dot{\varepsilon }}_{p}\right)\\ E=E_{0}\exp\left(\frac{Q_{E}}{RT}\right) \end{array}\right.$$

where ${\dot{\varepsilon }}_{t}$ is total strain rate, *E* means the Young’s modulus, and $E_{0}(\mathrm{GPa}),$ and $Q_{E}(\mathrm{kJ}/\mathrm{mol})$ are material constants, respectively.

### 2.2. Microstructural Evolution

From Equations (1)–(5), it is evident that the flow stress is governed by multiple microscopic parameters, necessitating a detailed analysis of their dynamic evolution.

#### 2.2.1. Dislocation Evolution

During thermal deformation, the dislocation density is influenced by work hardening (WH), dynamic recovery (DRV), and dynamic recrystallization (DRX), which can be expressed as follows [52]:

(8) $$\dot{\rho }={\dot{\rho }}_{\mathrm{WH}}-{\dot{\rho }}_{\mathrm{DRV}}-{\dot{\rho }}_{\mathrm{DRX}}$$

Work hardening, a phenomenon resulting from the accumulation of dislocations during plastic deformation, is directly correlated with the increase in dislocation density. This process is significantly influenced by strain rate and the mean free path of dislocations, which is determined by the obstacles impeding their motion. The relationship between these factors can be quantitatively described by [44]:

(9) $${\dot{\rho }}_{\mathrm{WH}}=\frac{M}{b}\left(\frac{\sqrt{\rho }}{k_{w}}+\frac{1}{d}+\sqrt{\frac{3f_{\delta }}{2\pi }}\frac{1}{r_{\delta }}\right){\dot{\varepsilon }}_{p}$$

where *k*_w_ is a material constant.

The DRV behavior leads to a decrease in the dislocation density, which can be represented by [52]:

(10) $${\dot{\rho }}_{\mathrm{DRV}}=k_{v}{\dot{\varepsilon }}_{p}^{n_{v}}\rho \exp\left(-\frac{Q_{v}}{RT}\right)$$

where $k_{v}$, $n_{v}$, and $Q_{v}(\mathrm{kJ}/\mathrm{mol})$ are material constants.

According to the previous report [39], the process of dislocation density consumption during the DRX behavior and δ phase dissolution is similar, i.e.,

(11) $${\dot{\rho }}_{\mathrm{DRX}}=\frac{k_{1}\left(\rho -\rho_{0}\right)}{{\left(1-X-X_{\delta }\right)}^{k_{2}}}\left(\dot{X}+{\dot{X}}_{\delta }\right)$$

where *X* (%) means the DRX fraction, $X_{\delta }$ (%) represents the dissolution fraction of δ phase, and *k*_1_ and *k*_2_ are material constants.

#### 2.2.2. DRX Fraction Evolution

Referring to related research [51,53], the evolution of the DRX fraction can be represented by the following:

(12) $$\left\{\begin{array}{l} \dot{X}=v\dot{N}{\left(1-X\right)}^{m_{1}}u\left(\varepsilon -\varepsilon_{c}\right)\\ v=\frac{1}{2}\mu b^{2}\rho \cdot \frac{w_{0}}{T}\exp\left(-\frac{Q_{w}}{RT}\right)\\ \dot{N}=N_{0}{\dot{\varepsilon }}_{p}^{m_{2}}\exp\left(-\frac{Q_{N}}{RT}\right)\exp\left(l_{\delta }\frac{f_{\delta }}{r_{\delta }}\right) \end{array}\right.$$

where *v* represents the grain boundaries migration speed, $\dot{N}$ means the nucleation rate, *m*_1_, *m*_2_, *w*_0_ (K/GPa), *N*_0_, $Q_{w}(\mathrm{kJ}/\mathrm{mol})$, $Q_{N}(\mathrm{kJ}/\mathrm{mol})$, and $l_{\delta }\left(\mu m\right)$ are material constants, while the function $u\left(x\right)=\left\{\begin{array}{ll} x, & x>0\\ 0, & x\leq 0 \end{array}\right.$.

Taking into account the effect of δ phase, the critical strain of DRX can be expressed as [54]:

(13) $$\varepsilon_{c}=m_{3}{\dot{\varepsilon }}^{m_{4}}\exp\left(\frac{Q_{\varepsilon }}{RT}\right)\exp\left(-V_{\delta }f_{\delta }\right)$$

where *m*_3_, *m*_4_, and $Q_{\varepsilon }(\mathrm{kJ}/\mathrm{mol})$ are material constants, $V_{\delta }$ is an influence coefficient of δ phase.

#### 2.2.3. Grain Size Evolution

The evolution of grain size during thermal deformation is primarily influenced by DRX [55], while the δ phase also has a significant effect. Therefore, the relationship can be expressed as [44]:

(14) $$\dot{d}=k_{d}\exp\left(-\frac{Q_{d}}{RT}\right){\left(\frac{E_{d}}{d}-\frac{3E_{\delta }f_{\delta }}{2r_{\delta }}\right)}^{\varphi_{1}}-\varphi_{2}\dot{X}d^{\varphi_{3}}$$

where $\varphi_{1}$, $\varphi_{2}$,$\varphi_{3}$, $k_{d}$, and $Q_{d}(\mathrm{kJ}/\mathrm{mol})$ are material constants, while $E_{d}$ and $E_{\delta }$ are the influence coefficients of grain boundary energy and phase boundary energy, respectively.

#### 2.2.4. Dissolution of δ Phase

According to the findings of He et al. [56], the dissolution kinetics of the δ phase are governed by several key factors, including strain, strain rate, temperature, and δ phase content. Moreover, the dissolution rate is further correlated with the maximum principal strain ($\varepsilon_{1}$) [20], i.e.,

(15) $$\left\{\begin{array}{l} {\dot{X}}_{\delta }=k_{\delta }{\left(\frac{\varepsilon +\left|\varepsilon_{1}\right|}{2}\right)}^{n_{1}}{\dot{\varepsilon }}^{n_{2}}N_{\delta }\\ N_{\delta }=\left(1-X_{\delta }\right)\exp\left(-n_{3}f_{\delta }\right)\\ k_{\delta }=k_{\delta 0}\exp\left(-\frac{Q_{k}}{RT}\right) \end{array}\right.$$

where $k_{\delta 0}$, $n_{1}$, $n_{2}$, $n_{3}$, and $Q_{k}(\mathrm{kJ}/\mathrm{mol})$ are material constants, $X_{\delta }$ means the dissolution ratio of δ phase, and $N_{\delta }$ denotes the influence term of the remaining δ phase.

#### 2.2.5. Damage Fraction Evolution

The Oyane model is an effective damage mechanics framework grounded in micromechanics, which can be expressed as [57]:

(16) $$\frac{\partial D}{\partial \bar{\varepsilon }}=1+A\frac{\sigma_{H}}{\bar{\sigma }}$$

where $\bar{\sigma }$ (MPa) is equivalent stress, $\bar{\varepsilon }$ is equivalent strain, $\sigma_{H}$ (MPa) represents the hydrostatic stress, and *A* is a material constant with the value of 0.424 [58].

It is widely accepted that the fraction of damage evolves continuously during deformation, and fracture occurs once the fraction reaches a critical threshold [59]. However, as shown in Figure 1, the evolution of damage varies under different stress states. Therefore, the accurate modeling of damage accumulation necessitates the incorporation of additional factors beyond the equivalent stress alone.

By the chain rule, the increment of damage over time can be obtained by the following:

(17) $$\dot{D}=\frac{\partial D}{\partial \varepsilon }\cdot \frac{d\varepsilon }{dt}=\dot{\varepsilon }\frac{\partial D}{\partial \varepsilon }$$

Referring to the viewpoints of Wen et al. [60], the damage evolution is related to strain, strain rate, temperature, and the existing damage fraction, while the second phase represents the initial nucleation site of the damage. Therefore, the damage fraction can be updated as follows:

(18) $$\left\{\begin{array}{l} \dot{D}={\dot{D}}_{N}+{\dot{D}}_{G}-{\dot{D}}_{R}\\ {\dot{D}}_{N}=\eta_{1}f_{\delta }{\dot{\varepsilon }}^{q_{1}}\exp\left(\eta_{2}\varepsilon \right)\exp\left(-\frac{Q_{D}}{RT}\right)\\ {\dot{D}}_{G}=\eta_{3}\left(1-D\right){\dot{\varepsilon }}^{q_{2}}\\ {\dot{D}}_{R}=\eta_{4}D \end{array}\right.$$

where ${\dot{D}}_{N}$, ${\dot{D}}_{G}$, and ${\dot{D}}_{R}$ represent the nucleation, growth, and restoration term of damage evolution, respectively. $q_{1}$, $q_{2}$, $\eta_{1}$, $\eta_{2}$, $\eta_{3}$, $\eta_{4}$, and $Q_{D}(\mathrm{kJ}/\mathrm{mol})$ are material constants.

Considering the direction of maximum principal stress, based on Equations (16)–(18), the evolution of the damage fraction can be rewritten as follows:

(19) $$\dot{D}=\left[\eta_{1}f_{\delta }{\dot{\varepsilon }}^{q_{1}}\exp\left(\eta_{2}\varepsilon \right)\exp\left(\frac{Q_{D}}{RT}\right)+\eta_{3}\left(1-D\right){\dot{\varepsilon }}^{q_{2}}-\eta_{4}D\right]\left(\frac{u\left(\sigma_{1}\right)}{\sigma_{u}}+A\frac{\sigma_{H}}{\bar{\sigma }}\right)$$

where $\sigma_{1}$ (MPa) means the maximum principal stress. $\sigma_{u}$ (MPa) represents the ultimate tensile strength.

### 2.3. Identification of Model Parameters

Based on the experimental data, a comprehensive dataset comprising 750 samples was generated, with 10 strain-equally spaced samples collected from each true stress–strain (TSS) curve under various processing conditions. Specific strain values were selected at intervals of Δ*ε* = 0.035 for tension (*ε* = 0.035, 0.07, …, 0.35) and Δ*ε* = 0.08 for compression (*ε* = 0.08, 0.016, …, 0.80), ensuring the uniform coverage of the plastic deformation regime (elastic regions excluded). Utilizing the MATLAB (2022b) Nonlinear Optimization Toolbox with a maximum iteration limit of 1000. The material constants were systematically calibrated. Table 1 summarizes the optimal combinations of these calibrated material constants.

## 3. Analysis and Discussion on the Developed Model

In this section, the accuracy of the unified constitutive model for a nickel-based superalloy is rigorously validated. Subsequently, the established model is employed to predict key microstructure parameters under diverse stress–strain conditions. The variation patterns of these parameters are then systematically analyzed to provide deeper insights into the material behavior.

### 3.1. Prediction and Validation of the Developed Constitutive Models

**Figure 2:**
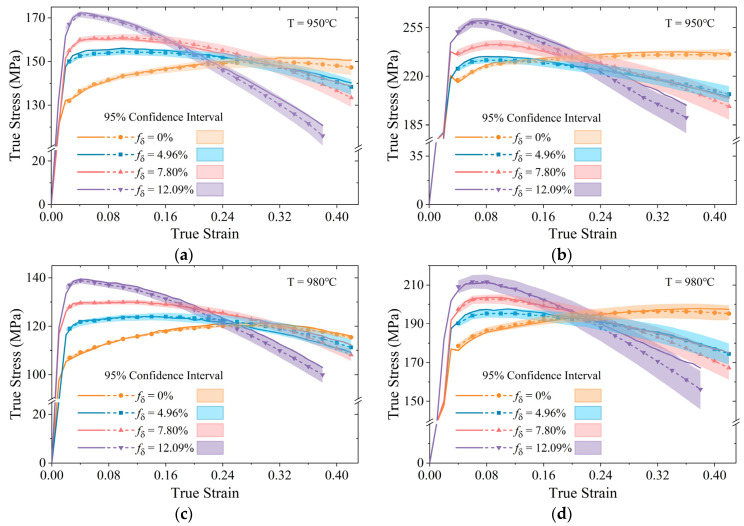

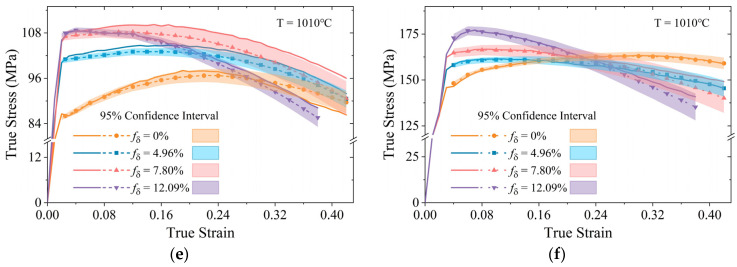
Comparison of predicted and measured TSS curve under tensile stress with different strain rates: (**a**,**c**,**e**) $\dot{\varepsilon }$ = 0.001 s^−1^; (**b**,**d**,**f**) $\dot{\varepsilon }$ = 0.01 s^−1^.

**Figure 3:**
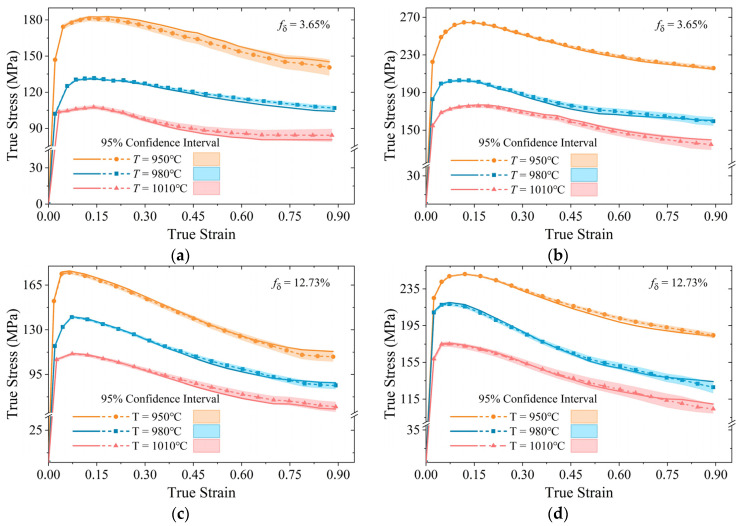

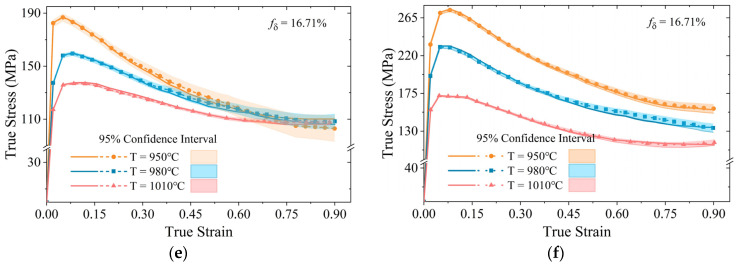
Comparison of predicted and measured TSS curve under compressive stress with different strain rate: (**a**,**c**,**e**) $\dot{\varepsilon }$ = 0.001 s^−1^; (**b**,**d**,**f**) $\dot{\varepsilon }$ = 0.01 s^−1^.

Figure 2 and Figure 3 illustrate the predicted TSS curves for a nickel-based superalloy subjected to uniaxial tensile stress or compressive stress, respectively. The experimental data for the rheological curves, as reported by Lin et al. [42] and Chen et al. [43], are depicted by smooth solid lines, while the model predictions are represented by dashed lines with markers. Different colors indicate varying initial δ phase contents. The shaded regions denote the 95% confidence intervals of the predicted data, which were calculated using the Locally Weighted Scatterplot Smoothing (LOWESS) method. These intervals provide a statistical measure of the uncertainty associated with the predictions.

As illustrated in Figure 2, at a consistent temperature and strain rate, the initial δ phase content exerts a significant influence on the morphology of rheological curves. At relatively low strain (ε<0.2), a higher initial δ phase content correlates with increased true stress, demonstrating the strengthening effect of phases. However, once the strain surpasses a certain threshold (ε>0.3), materials with a higher initial δ phase content display a significant reduction in true stress, suggesting a diminution in ductility and an escalation in internal microcracking.

Meanwhile, a higher initial δ phase content leads to a broader stress confidence interval for true stress predictions under identical deformation conditions, reflecting the increased uncertainty in the material rheological behavior. Under specific conditions (e.g., 1010 °C and 0.001 s^−1^), the measured results at elevated strains may deviate beyond the 95% confidence interval of the predicted values. Nevertheless, the predictive accuracy of the proposed model demonstrates significant improvement over previous models [42].

In contrast to Figure 2, the rheological curves in Figure 3 presented no yield phenomena. Additionally, the hardening and softening effects observed in Figure 3 are more pronounced, indicating the obvious microstructure evolution and enhanced compressive plasticity.

Comparing the pairs of images in Figure 3, specifically (a, c, e) and (b, d, f), it is evident that the peak values of the rheological curves exhibit no significant variations under identical conditions and varying initial δ phase contents. This suggests that the initial δ phase content has a limited influence on the maximum flow stress during hot deformation. Furthermore, with increasing initial δ phase content and true strain, the stress values at different deformation temperatures tend to converge. This convergence indicates that the second-phase strengthening effect under compressive stress is relatively minor, likely due to the dissolution of δ phase and the saturation of DRX [37]. Notably, as the true strain increases, the softening effect becomes more pronounced with higher initial δ phase content. This observation underscores the role of the δ phase in influencing the recrystallization behavior, particularly promoting DRX and contributing to an overall softening mechanism.

### 3.2. Evaluation and Comparison of Prediction Accuracy

In order to quantitatively assess the predictive performance of the proposed model, two evaluation metrics shown in Equations (20) and (21) are utilized. The Average Absolute Relative Error (*AARE*) emphasizes the relative error between predicted and actual values, while the Coefficient of Determination (*R*^2^) measures how effectively the model predicts future outcomes. By quantifying these two metrics, the accuracy and reliability of the rheological curve prediction model for nickel-based superalloys can be effectively assessed.

(20) $$AARE=\frac{1}{N}\sum_{i=1}^{N}\left|\frac{y_{i}-{\hat{y}}_{i}}{y_{i}}\right|\times 100\%$$

(21) $$R^{2}=1-\frac{\sum_{i=1}^{N}{\left(y_{i}-{\hat{y}}_{i}\right)}^{2}}{\sum_{i=1}^{N}{\left(y_{i}-{\bar{y}}_{i}\right)}^{2}}$$

where $y_{i}$ represents the predicted value, ${\hat{y}}_{i}$ means the experimental value, ${\bar{y}}_{i}$ is the average of experimental value.

**Figure 4:**
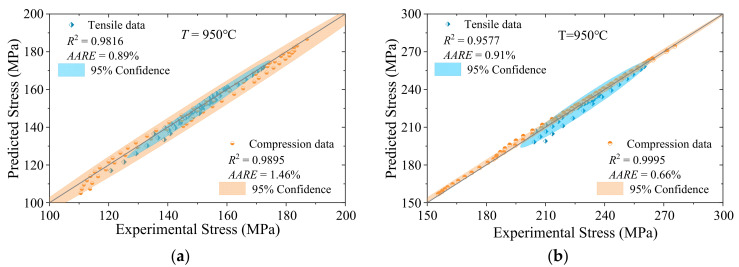

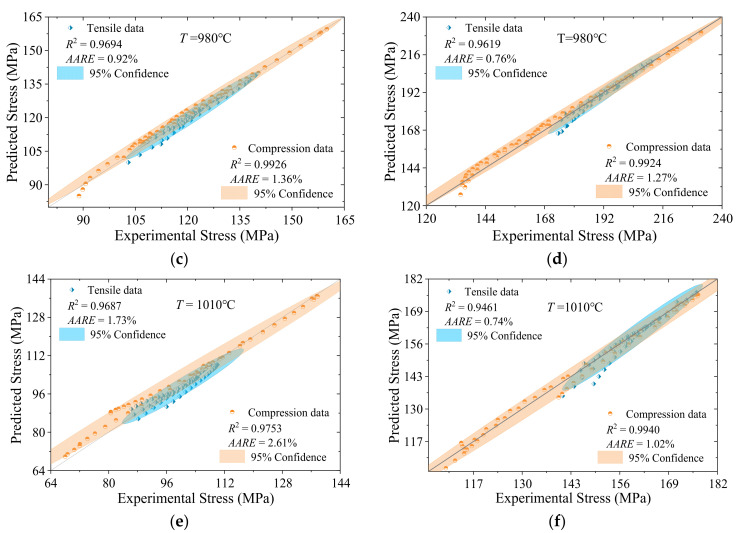
Correlations between the measured and predicted stresses with different strain rate: (**a**,**c**,**e**) $\dot{\varepsilon }$ = 0.001 s^−1^; (**b**,**d**,**f**) $\dot{\varepsilon }$ = 0.01 s^−1^.

Figure 4 shows the correlations between the predicted and measured stresses presented in Figure 2 and Figure 3. The values of tensile stress are indicated by blue diamond markers, while the values of compressive stress are shown as red circular markers. The shaded area represents the confidence ellipse, which is derived from the linear fitting of the data and corresponds to a 95% confidence level.

As illustrated in Figure 4, the *AARE*s under all conditions are less than 3%, indicating an ideal predictive accuracy. Under varying temperature conditions, the *R*^2^ values under compressive stress are higher than those under tensile stress. However, the confidence ellipses are generally wider in most cases, suggesting that the rheological range of the material under compressive stress is larger. Nevertheless, the uncertainty remains stable, further highlighting the robust predictive capability of the model.

### 3.3. Effects of Initial δ Phase Contents on Microstructure Evolution

**Figure 5:**
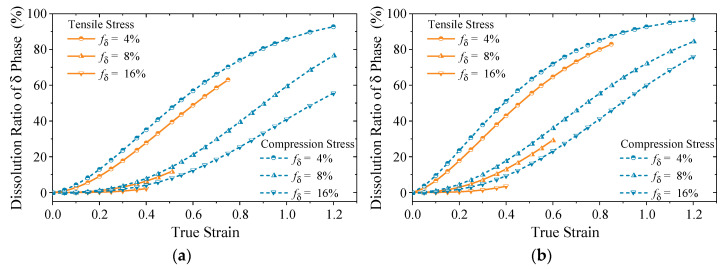
Dissolution ratio of δ phase at: (**a**) *T* = 950 °C, $\dot{\varepsilon }$ = 0.001 s^−1^; (**b**) *T* = 1010 °C, $\dot{\varepsilon }$ = 0.01 s^−1^.

**Figure 6:**
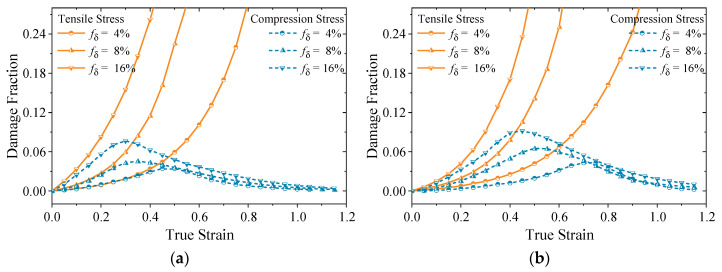
Damage fraction at (**a**) *T* = 950 °C, $\dot{\varepsilon }$ = 0.001 s^−1^; (**b**) *T* = 1010 °C, $\dot{\varepsilon }$ = 0.01 s^−1^.

Using the validated model, the predictions of phase dissolution kinetics and damage volume fractions have been systematically conducted under diverse microscope stress states and varying initial δ phase contents. The results for two distinct conditions are presented in Figure 5 and Figure 6, respectively.

Figure 5 illustrates that under varying conditions, the dissolution ratio of the δ phase follows a sigmoid trend as strain increases. At a constant true strain, a lower initial δ phase content corresponds to a higher dissolution rate of the δ phase. Additionally, an increase in deformation temperature accelerates the attainment of dissolution equilibrium. A comparison between the solid and dashed lines indicates that under identical conditions, the dissolution rate of the δ phase under tensile stress is greater than that under compressive stress. This phenomenon can be attributed to the enhanced bending of the δ phase under compressive stress, which promotes the dissolution. This observation is consistent with the trend depicted in Figure 3.

Figure 6 shows that the damage fraction increases across varying stress states when the true strain is small. This phenomenon indicates that small strains can result in an increase in the volume fraction of damage even under compressive stress. Moreover, at low strain rates and with small initial δ phase content, the differences among various stress states with small strain are negligible. However, as the strain continues to increase beyond a critical threshold, compressive stress promotes pore closure, ultimately resulting in a damage-free state. In contrast, tensile stress induces a rapid expansion in pore volume and aggregation, leading to crack formation. Furthermore, an increase in the initial δ phase content accelerates the crack propagation rate, significantly impairing the material plasticity.

Figure 5 and Figure 6 reveals a significant correlation between the increase in damage fraction and the dissolution behavior of δ phase under tensile stress conditions. The damage evolution exhibits a strong dependence on the dissolution kinetics of δ phase, where limited δ phase dissolution at low strain corresponds to a gradual increase in damage fraction. In contrast, the enhanced δ phase dissolution at higher strains accelerates damage nucleation and propagation, leading to a more pronounced increase in damage fraction. Under compressive stress conditions, a complex interplay between the void formation induced by δ phase dissolution and concurrent void closure mechanisms is observed. This competing process establishes a dynamic equilibrium state in the damage fraction, demonstrating the stress–state dependent damage evolution characteristics in this material system.

## 4. Application of the Constitutive Model in Forging Processes

### 4.1. The FE Integrated Computational Method

To maximize the practical utility of the model, the unified constitutive model should be integrated into a user-defined subroutine within FE software (ABAQUS 2020), enabling seamless and comprehensive computational simulations. This approach is expected to be broadly applicable for the simulation and prediction of thermal processing behaviors in the same alloy system.

**Figure 7:**
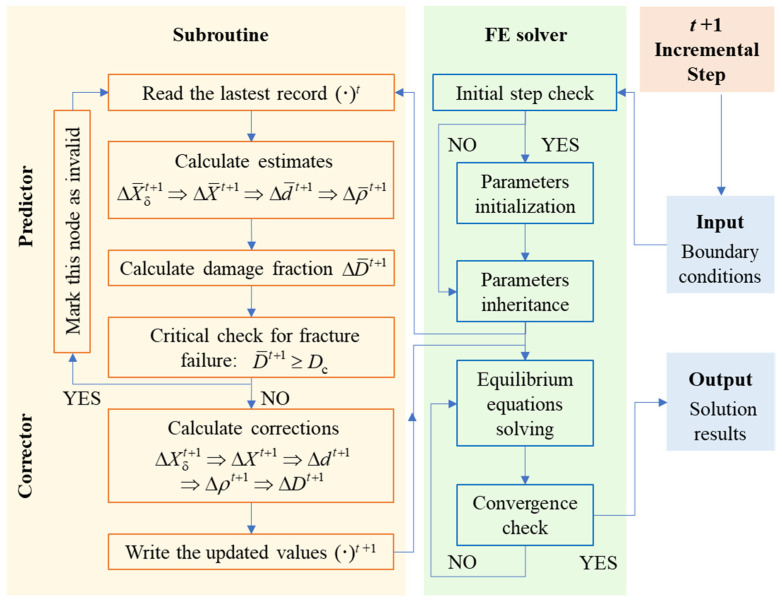
An integrated computational framework of the unified constitutive model and FE method.

Figure 7 illustrates the integrated computational framework proposed based on the unified constitutive model developed in Section 2. From Equations (14) and (18), it is evident that the computation involves first-order partial differential equations. Therefore, an explicit-implicit method is employed for iterative solutions. The sequence of parameter solutions is as follows: increment of the δ phase dissolution rate, increment of the dynamic recrystallization volume fraction, increment of the average grain size, increment of dislocation density, and increment of damage. If the damage fraction of the integration point exceeds a critical value, it is marked as failure and stress-free.

**Figure 8:**
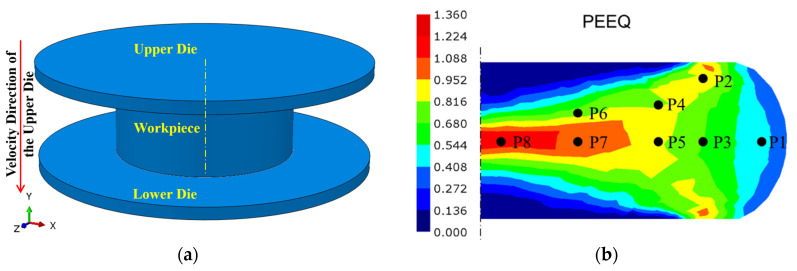
The integrated computational FE model to be verified: (**a**) Schematic of a 3-dimensional geometric model. (**b**) The selected critical regions over the obtained contour plots of plastic equivalent strain.

Figure 8 presents a schematic diagram of a practical application example. The FE model is constructed to scale with the actual object, utilizing a billet made from a nickel-based superalloy ingot with a diameter of 233 mm and a height of 140 mm. The reduction in height is 60 mm, and the total time measured according to the actual forging process is 34.8 s, resulting in an overall average strain rate of approximately 0.01 s^−1^.

The die temperature was maintained at 500 °C, with a thermal convection coefficient of 0.02 N/s/°C/mm. The friction coefficient was 0.1 [31]. The thermal and mechanical parameters of the nickel-based superalloy, with the following chemical composition (wt.%), 52.82 Ni, 18.96 Cr, 5.23 Nb, 3.01 Mo, 1.00 Ti, 0.59 Al, 0.01 Co, 0.03 C, and balance Fe, are presented at various temperatures shown in Table 2. The simulated plastic equivalent strains are illustrated in Figure 8b. To facilitate a comparison with subsequent experimental results, eight critical positions exhibiting different states within the deformation area were selected to extract the calculation results.

### 4.2. Experimental Validatio

The forgings used in this experiment were supplied by Fushun Special Steel Co., Ltd. in Fushun, China, and were produced under the same batch and heat treatment conditions as those in a previous study [62]. The pre-forging heat treatment parameters were selected based on the optimal combination of processes identified in earlier research [63], which involved maintaining the material at 980 °C for 120 min.

**Figure 9:**
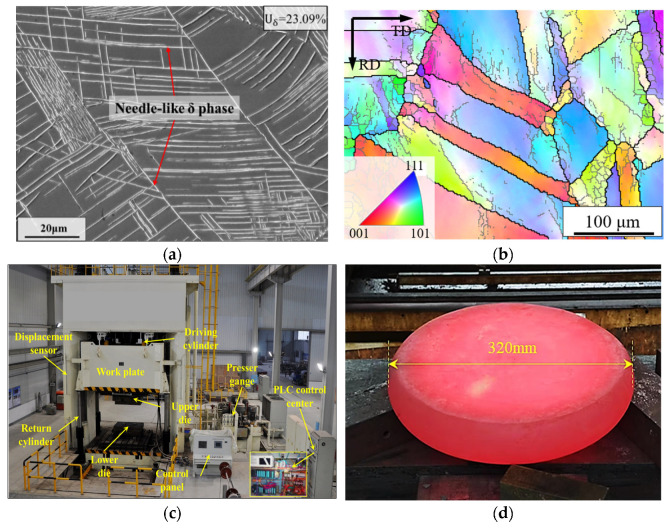
Results of the forging experiment: (**a**) SEM image of the initial microstructure; (**b**) EBSD map of the initial microstructure; (**c**) hydraulic forging equipment; (**d**) hot disk forging.

Figure 9a,b presents the scanning electron microscope (SEM) image and Electron Backscatter Diffraction (EBSD) map of the initial microstructure, respectively. The initial content of the δ phase was statistically determined as 23.09% with the software named Image Pro Plus 7.0, while the initial grain size was measured as 28.2 μm. These quantitatively characterized microstructural parameters were subsequently input into the FE software for parameter initialization.

The actual image of forging produced by the hydraulic forging equipment (Figure 9c) is presented in Figure 9d, with the initial size of 233 mm in diameter and 140 mm in height. The samples were quenched in brine until they reached room temperature and then sectioned using a DK-7730 wire-cut electric discharge machine (Made in Jangsu Province, China), in order to preserve the original microstructure after forging. Samples from the non-deformation zone were taken as the initial microstructure of forging, and from the selected eight inspection areas, samples with Φ8 mm × 3 mm were cut perpendicular to the longitudinal section. The subsequent treatment plans and observation methods were consistent with those employed in a previous report [64].

## 5. Verification and Analysis of the Application Case with Disk Forging

**Figure 10:**
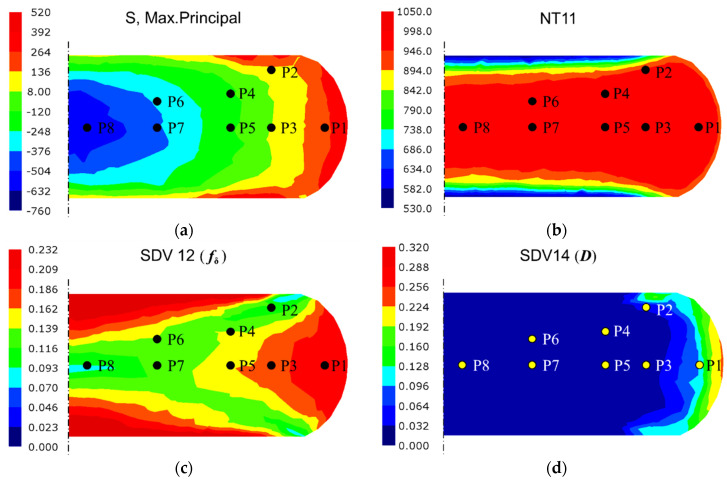
Contour plots of integrated computational results at the last step: (**a**) maximum principal stress; (**b**) temperature; (**c**) fraction of δ phase; (**d**) fraction of damage. (P1–P8 are the selected feature regions).

Figure 10 illustrates the contour plots of FE simulation results obtained with the integrated computational framework at the last time step. The plots encompass two macroscopic parameters (maximum principal stress and temperature) and two microscopic parameters (δ phase volume fraction and damage volume fraction) across varying regions. As demonstrated in Figure 10a, regions P1 and P2 exhibit positive maximum principal stress values, resulting in a progressive increase in damage volume fraction. This observation is in excellent agreement with the damage evolution characteristics presented in Figure 10d. Furthermore, the absolute magnitude of the maximum principal stress exceeds the rheological stress under equivalent uniaxial compression conditions by an order of magnitude, suggesting the presence of significant hydrostatic stress components and enhanced material flow characteristics [65]. The temperature contour plot in Figure 10b shows minimal thermal elevation within forging, which corresponds with the low strain rate.

Figure 8b and Figure 10c demonstrate that the residual δ phase fraction exhibits a strong dependence on the synergistic interaction between true strain and true stress fields. Specifically, while regions P3, P4, and P6 share comparable plastic strain levels around 0.7, region P3 maintains a higher residual δ phase fraction due to its lower Von Mises equivalent stress. This stress–strain dependent behavior is further evidenced by a comparison between regions P4 and P5, where similar stress magnitudes (Δ*σ*_1_ < 10 MPa) but differing strain levels result in a 16.3% reduction in δ phase retention in region P5. Region P1, characterized by tensile-dominated stress state and medium plastic strain (*ε*_p_ = 0.43), exhibits the least δ phase dissolution, corroborating the phenomenological observations in Figure 5. Furthermore, the central P2 and P8 regions, subjected to extensive plastic deformation (ε_p_ > 0.9), exhibit pronounced δ phase dissolution, with the fraction reductions exceeding 30%, highlighting the critical role of strain magnitude in phase transformation kinetics.

**Figure 11:**
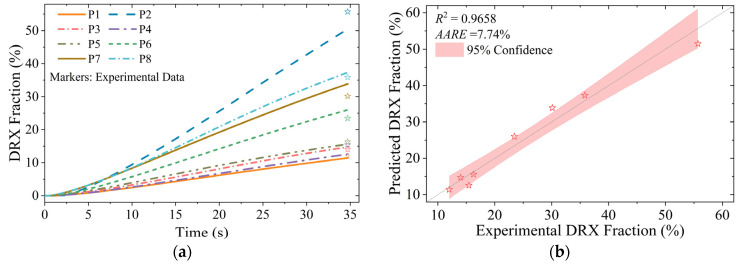
Prediction results and verification of DRX fraction evolution: (**a**) predicted values over time; (**b**) comparison of experimental and predicted values.

**Figure 12:**
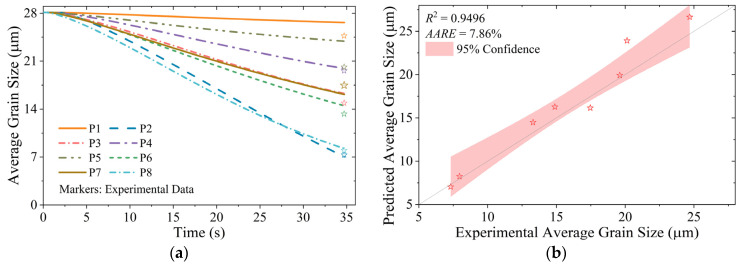
Prediction results and verification of grain size evolution: (**a**) predicted values over time; (**b**) comparison of experimental and predicted values.

The temporal evolution of DRX fraction and average grain size predicted by the integrated computational framework is systematically presented in Figure 11 and Figure 12, respectively. Experimental validation data points are superimposed on the simulation results, demonstrating a good agreement between predictions and measurements. The AARE for DRX fraction prediction is 7.74%, while the average grain size estimation shows a relative error of 7.86%, both within acceptable limits of ±10% for engineering applications.

Region P1 exhibits the lowest DRX volume fraction among all analyzed zones, attributable to its limited plastic strain accumulation and relatively low thermal activation. So, the grain refinement (Δ*d* < 5 μm) is minimal. During the initial deformation stage (*t* < 5 s), the microstructure evolution in region P2 closely parallels that of region P1. However, with progressive deformation, region P2 experiences accelerated strain accumulation coupled with the lowest thermal conditions (*T* < 950 °C), which effectively suppresses grain growth kinetics. The combined effects of reduced δ phase dissolution rate and increased density of potential nucleation sites promote intensive discontinuous DRX in the following deformation stage (*t* > 15 s). Consequently, region P2 achieves the most refined microstructure, with an average grain size of 7.3 μm, representing a 73.9% reduction compared with the initial grain structure.

Regions P3, P4, and P5 demonstrate comparable deformation characteristics, with equivalent strain ranges ($\bar{\varepsilon }$ = 0.8~1.0) and temperature levels (*T* = 970~980 °C), resulting in similar DRX volume fractions (*X* = 12~15%). However, the lower absolute value of maximum principal stress in region P3 (approximately 120 MPa lower than those in regions P4 and P5) and higher dissolution ratio of δ phase enhance grain refinement, yielding a finer microstructure with an average grain size of 14.9 μm, compared with 19.6 μm in region P4 and 20.1 μm in region P5.

The central zones, regions P6, P7, and P8, exhibit a progressive increase in both strain ($\bar{\varepsilon }$ = 0.7~1.1) and stress ($\left|\sigma_{1}\right|$ = 330~590 MPa), leading to corresponding increases in DRX fraction (*X* = 23.4~35.8%). The relatively lower temperature in region P6 (*T* ≈ 965 °C) results in a finer grain with average grain sizes of 13.3 μm, compared with 17.5 μm in region P7 (*T* ≈ 985 °C). Region P8 is subjected to the most significant deformation conditions and exhibits the highest δ phase dissolution ratio. As a result, it demonstrates the most extensive continuous DRX behavior, achieving an average grain size of 7.9 μm (Supplementary EBSD orientation maps are presented in Appendix A).

**Figure 13:**
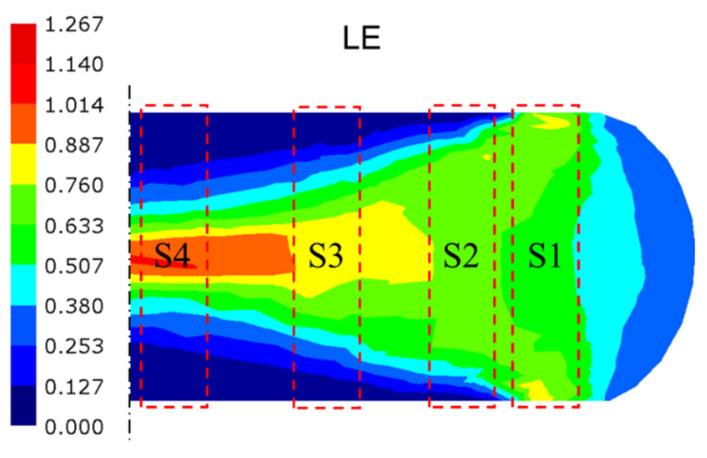
Sampling position for uniaxial tensile specimens, with the background depicting the logarithmic strain.

**Figure 14:**
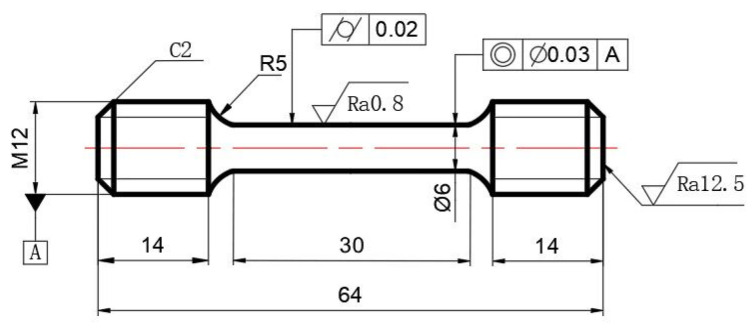
Dimensions of the specimens for tensile tests (unit: mm).

**Figure 15:**
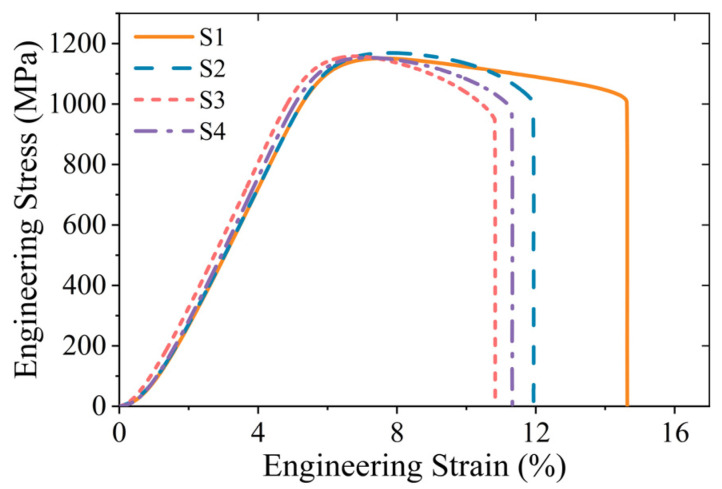
Engineering stress–strain curves of samples.

Additionally, cylindrical samples were extracted from the locations indicated in Figure 13. After machining, these samples were fabricated into tensile test specimens, whose dimensional specifications and tolerance ranges are detailed in Figure 14. The samples are labeled S1 to S4 correspond to the mechanical properties of P3, P5, P7, and P8, respectively. In Figure 15, the four curves exhibit continuous yielding behavior without a distinct yield point. Consequently, the yield strength was determined using the 0.2% offset method, corresponding to the engineering stress at 0.2% permanent strain [66]. The key performance indicators are systematically evaluated and compared in Table 3.

The mechanical property evaluation reveals that the as-forged disk fails to meet the required standard [67], necessitating comprehensive optimization of post-forging heat treatment parameters. As shown in Table 3, the tensile strengths of different specimens exhibit minimal variation. Correlative analysis of Figure 10c, Figure 11 and Figure 12 reveals that regions with lower residual δ phase content (e.g., P8) display refined grain sizes after substantial plastic deformation, whereas regions with higher δ phase content (e.g., P5) show coarser grains. These observations suggest a mutual compensation effect between precipitation strengthening and grain boundary strengthening mechanisms.

The tensile behavior reveals significant anisotropy in plastic properties across different regions. Specifically, S1 specimen exhibits the highest elongation but the lowest reduction in area, suggesting that the region P3 demonstrates high deformation uniformity but overall poor plasticity. When correlated with the microstructural observations presented in Figure 5b, it can be attributed to the combined effects of the relatively small true strain and the higher remaining δ phase content, which collectively influence the deformation mechanisms and damage tolerance characteristics.

The performance of the S2 specimen is closer to the inspection standard values (within 1% deviation), indicating that the thermomechanical processing conditions in region P5 achieve optimal microstructure characteristics. These findings provide critical empirical data for optimizing final forging die geometry and processing parameters in industrial application. While specimens S3 and S4 exhibit substandard elongation values, they demonstrate an exceptional reduction in area, indicating pronounced necking behavior and localized plasticity. The corresponding regions P7 and P8 show enhanced local formability but suffer from the uniformity along the height direction, consistent with the predicted strain distribution patterns in computational simulations. These results further validate the accuracy of the model’s predicted outcomes.

## 6. Conclusions

Existing constitutive models inadequately capture the mechanisms of microstructure evolution during hot deformation of nickel-based superalloys. To address this gap, an advanced microstructure-based unified constitutive model that integrates macroscopic stress–strain effects with multiscale descriptors is developed. Additionally, a FE-based computational framework is established and validated through disk forging experiments. Key findings include the following:

(1) The proposed model can quantitatively capture the evolution of dislocation density, DRX volume fraction, average grain size, δ phase dissolution ratio, and damage volume fraction. Validation against uniaxial tension and compression rheological curves demonstrated high accuracy, with an AARE of less than 3% and the R^2^ greater than 0.95.
(2) Mechanistic analysis revealed that the dissolution of the δ phase exhibits strain-dependent kinetics, with a dissolution rate that is faster under compression compared with tension. Additionally, tensile stress induces damage accumulation that is proportional to the initial δ phase content, while compressive stress suppresses damage formation below a critical strain threshold.
(3) The implementation of FE software facilitated the analysis of eight critical regions in disk forging. The predicted recrystallization fraction and average grain size maintained an AARE of less than 8%. Maximum recrystallization and grain refinement occurred in near-surface regions that experienced severe plastic deformation.
(4) High-temperature tensile tests conducted at 650 °C across four regions demonstrated less than 5% variation in strength but over 40% difference in plasticity. Subsurface areas exhibited optimal ductility, achieving 14.61% elongation, while sub-central zones experienced constrained deformation. These findings validate the model’s predictive accuracy and highlight the industrial applicability of the computational method.

## Figures and Tables

**Table 1:** The optimized material constants.

Constants	Value	Constants	Value	Constants	Value
$\alpha_{\delta 0}$	0.035	$k_{\delta 0}$	−334.2	$n_{3}$	1.450
$\alpha_{t0}$	1.928 × 10^−9^	$k_{v}$	6.204 × 10^3^	$n_{v}$	0.843
$E_{0}$	0.661	$k_{w}$	0.051	$\eta_{1}$	0.078
$E_{d}$	291.5	$m_{1}$	0.545	$\eta_{2}$	2.054 × 10^−3^
$E_{\delta }$	438.2	$m_{2}$	0.360	$\eta_{3}$	0.433
$\varphi_{1}$	2.786	$m_{3}$	6.091 × 10^−3^	$\eta_{4}$	0.067
$\varphi_{2}$	1.509	$m_{4}$	0.153	$Q_{D}$	5.882 × 10^4^
$\varphi_{3}$	1.114	$N_{0}$	1.722 × 10^4^	$Q_{d}$	3.319 × 10^5^
$k_{1}$	6.843	$n_{0}$	4.026	$Q_{\delta }$	8.562 × 10^3^
$k_{2}$	1.268	$n_{1}$	0.349	$Q_{E}$	5.375 × 10^4^
$k_{d}$	−2.470 × 10^−3^	$n_{2}$	0.761	$Q_{\varepsilon }$	1.624 × 10^5^
$Q_{k}$	2.694 × 10^4^	$Q_{v}$	9.837 × 10^4^	$\sigma_{0}$	59.12
$Q_{N}$	1.971 × 10^5^	$Q_{w}$	2.875 × 10^5^	$w_{0}$	7.556 × 10^12^
$Q_{n}$	3.806 × 10^4^	$q_{1}$	0.411		
$Q_{t}$	3.139 × 10^5^	$q_{2}$	1.029		

**Table 2:** The thermal and mechanical parameters at various temperatures [61].

Temperature/°C	500	700	900	1000	1100
Coefficient of heat conduction/N·s^−1^·°C^−1^	18.47	24.15	25.72	26.32	29.01
Young’s modulus/GPa	174	163	134	115	98
Heat capacity/N·mm^−2^·°C^−1^	4.28	5.02	5.35	5.53	5.69

**Table 3:** Uniaxial tensile properties of specimens at 650 °C.

Specimens	Yield Stress (MPa)	Tensile Stress (MPa)	Elongation (%)	Reduction in Area (%)
Standard [60]	1000	1170	12	15
S1	1016	1154	14.61	12.61
S2	1027	1167	11.92	15.70
S3	1019	1151	10.76	15.62
S4	1013	1149	11.25	20.08

**Figure A1:**
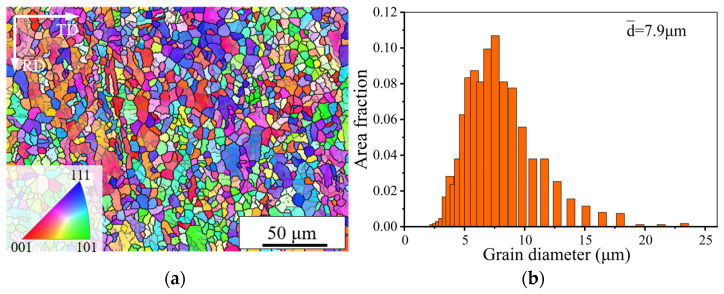
Post-forging EBSD analysis of the P8 region: (**a**) IPF orientation map; (**b**) grain size distribution.

## Data Availability

The original contributions presented in this study are included in the article. Further inquiries can be directed to the corresponding author.

## Appendix A

In Figure A1, the supplementary EBSD microstructural characterization of the P8 region confirms grain size validation through crystallographic orientation mapping and statistical distribution analysis.
